# The Renin–Angiotensin-Aldosterone System, Nitric Oxide, and Hydrogen Sulfide at the Crossroads of Hypertension and COVID-19: Racial Disparities and Outcomes

**DOI:** 10.3390/ijms232213895

**Published:** 2022-11-11

**Authors:** Tara Ranjbar, Palak P. Oza, Khosrow Kashfi

**Affiliations:** 1 Department of Molecular, Cellular and Biomedical Sciences, Sophie Davis School of Biomedical Education, City University of New York School of Medicine, New York, NY 10031, USA; 2 Graduate Program in Biology, City University of New York Graduate Center, New York, NY 10016, USA

**Keywords:** renin–angiotensin system, hypertension, nitric oxide, hydrogen sulfide, SARS-CoV-2, COVID-19, ACE2, RAAS inhibitors, health disparities

## Abstract

Coronavirus disease 2019 is caused by SARS-CoV-2 and is more severe in the elderly, racial minorities, and those with comorbidities such as hypertension and diabetes. These pathologies are often controlled with medications involving the renin–angiotensin–aldosterone system (RAAS). RAAS is an endocrine system involved in maintaining blood pressure and blood volume through components of the system. SARS-CoV-2 enters the cells through ACE2, a membrane-bound protein related to RAAS. Therefore, the use of RAAS inhibitors could worsen the severity of COVID-19’s symptoms, especially amongst those with pre-existing comorbidities. Although a vaccine is currently available to prevent and reduce the symptom severity of COVID-19, other options, such as nitric oxide and hydrogen sulfide, may also have utility to prevent and treat this virus.

## 1. Introduction

Coronavirus disease 2019 (COVID-19) has affected the world and is caused by severe acute respiratory coronavirus 2 (SARS-CoV-2). COVID-19 is primarily transmitted through respiratory droplets [1] and presents a broad range of symptoms. Clinical manifestations of this disease can present either asymptomatically or as flu-like symptoms, along with loss of taste and smell [2]. The most severe manifestations of the virus occur amongst the elderly and those with comorbidities, such as hypertension, diabetes mellitus, and other immunosuppressed states [3]. In addition, studies have shown that COVID-19 has disproportionately affected racial minorities [4,5]. The renin–angiotensin–aldosterone system (RAAS) is pathologically activated during these states. Usually, the RAAS plays a significant role in regulating blood pressure and volume and systemic vascular resistance [6]. Therefore, pathological activation of the RAAS results in excessive vasoconstriction and can cause hypertension and renal and cardiovascular disease [7]. One of the central RAAS regulators that plays a specific role in inducing endothelial dysfunction, vasoconstriction, and eventual fibrosis is angiotensin II [8]. To prevent angiotensin II from causing pathologies such as hypertension, angiotensin-converting enzyme (ACE) inhibitors and angiotensin receptor blockers (ARB) are prescribed [8].

It has been found that SARS-CoV-2 has access to the cells through an RAAS-related protein, ACE2. SARS-CoV-2 uses ACE2, which is present as a transmembrane and soluble form and acts as a receptor for the virus to enter the body [9]. ACE2 is often upregulated when comorbidities are present [10,11].

Even though people with comorbidities often use RAAS inhibitors to manage their underlying condition, their use could impact the severity of COVID-19 symptoms. Therefore, alternatives such as nitric oxide and hydrogen sulfide to treat and prevent COVID-19 are of significant interest.

In this article, we represent an overview of the role of RAAS in hypertension and the role of nitric oxide and hydrogen sulfide in COVID-19. We also look at racial disparities and view the clinical outcomes of COVID-19 associated with these disparities.

## 2. Renin–Angiotensin–Aldosterone System

The RAAS is a complex endocrine system that is critical in maintaining blood pressure through balancing electrolyte levels and blood volume [12]. The three main components of the hormonal system are renin, angiotensin II, and aldosterone [13]. Even though the baroreceptor mechanism is pertinent in managing short-term and sudden changes in blood pressure, the RAAS system is seen in more chronic, long-term scenarios [14]. In response to low blood pressure, the juxtaglomerular apparatus of the kidneys become activated and cleave the constitutively secreted proenzyme prorenin into its active form, renin [15]. Once renin is in the bloodstream, it is able to act on angiotensinogen, a protein that is produced in the liver and fat cells, and cleave it into angiotensin I [16]. Angiotensin I is then converted into angiotensin II through the angiotensin-converting enzyme (ACE), found primarily in the vascular endothelium of the lungs. Angiotensin II is considered to be the main RAAS agonist and acts on angiotensin II receptors, such as the AT1R, throughout the body [13]. Some of the roles of angiotensin II include acting as a vasoconstrictor through its effects on vascular smooth muscle, increasing heart rate and contractility through activation of the sympathetic nervous system, and stimulating the zona glomerulosa cells of the adrenal cortex to produce and secrete the hormone aldosterone [17]. The role of aldosterone is to activate the epithelial sodium channels (ENaC) found in the distal nephron and increase sodium and water reabsorption [18]. Through these mediators, the RAAS is able to regulate blood pressure. However, when the RAAS complex is pathologically activated, it results in excessive vasoconstriction, muscular hypertrophy within the cardiac and vascular systems, and fibrosis [19]. The imbalance and dysregulation of the RAAS is seen in various pathologies, such as hypertension, congestive heart failure, and diabetic nephropathy [20].

### Hypertension and RAAS Inhibitors

According to the World Health Organization, hypertension is one of the most common causes of premature death globally [21]. Worldwide, hypertension affects about one billion people [22]. Hypertension is dangerous because it causes a variety of complications, including heart disease, kidney dysfunction, stroke, and transient ischemic attacks. Since the net effects of the RAAS result in vasoconstriction, increased sodium and water retention, and increased myocardial contraction, all of these effects increase the effective circulating volume and thus increase the arterial blood pressure. When consistently activated, as seen in pathological situations, it results in hypertension [23]. For that reason, RAAS inhibitors, such as direct renin inhibitors, ACE inhibitors, ARBs, and aldosterone antagonists, are often prescribed in the treatment of hypertension [20].

## 3. COVID-19

Human coronaviruses commonly cause the common cold and are considered to cause relatively mild infections [24]. However, there have been three major coronavirus outbreaks within the last three decades [25], the latest being coronavirus disease 2019 (COVID-19), caused by severe acute respiratory coronavirus 2 (SARS-CoV-2). The novel SARS-CoV-2 was first isolated on 7 January 2020 [26] from a series of pneumonia cases in Wuhan City, China [27]. It has infected more than 595 million people across the globe and caused 6.4 million deaths [28]. According to the World Health Organization, the virus spreads through respiratory droplets. This includes droplets of saliva and or discharge through the nose when a person coughs, sneezes, or talks. The highest viral loads were found in the nose and throat after symptom onset [29]. The clinical manifestations of COVID-19 include but are not limited to fever, cough, fatigue, muscle aches, and loss of taste and smell [1]. Even though not everyone who contracts the COVID-19 virus is symptomatic, those who are asymptomatic are found to have similar viral load contents to those of symptomatic patients [29]. According to the Centers for Disease Control and Prevention (CDC), although COVID-19 is relatively mild in most cases, the severe and fatal cases are seen among the elderly and those with underlying medical conditions, including cardiovascular disease, hypertension, and diabetes.

COVID-19 presents with flu-like symptoms in most cases and, in more severe cases, can present with respiratory complications, cytokine storms, abnormal coagulopathy, and extensive endothelial damage. Between 5 and 20% of hospitalized COVID-19 patients require ICU-level care [30]. Severe cases can lead to acute respiratory distress syndrome (ARDS), associated with acute onset hypoxemia, edema, and bilateral lung infiltrate [31]. This respiratory compromise often necessitates the use of mechanical ventilation (MV), which comes with risks of secondary infection [32] and exacerbation of lung injury [33]. Severe COVID-19 complications largely arise due to a hyperinflammatory response [34,35], which leads to extensive inflammatory damage to the pulmonary and vascular systems [36,37]. In addition to respiratory complications and uncontrolled inflammation, COVID-19 is notable for resulting in elevated markers of coagulopathy and thrombotic complications, including pulmonary embolism and disseminated intravascular coagulation in severe cases [38,39,40,41,42]. These vascular complications are reflective of extensive damage to the vascular endothelium, which results in a shift to a prothrombotic state [43]. Endothelial damage results from both direct infection of endothelial cells by SARS-CoV-2 [44,45,46] and inflammatory damage from its characteristic ‘cytokine storm’ [47]. In fact, inflammation and endothelial dysfunction are common factors between various comorbidities that place individuals at risk of poorer outcomes from COVID-19, which include diabetes and hypertension [48,49,50,51]. Thus, successful management of COVID-19 requires antiviral therapy early in the course of the infection and attention to mitigating respiratory distress, resolving inflammation, and maintaining vascular integrity.

### COVID-19 and RAAS

People with diabetes or hypertension are often prescribed RAAS inhibitors to manage their diseases and prevent complications, such as diabetic nephropathy, cardiovascular disease, and mortality [52]. Even though these medications are beneficial for several indications, it is unclear whether or not they are exacerbating harm in COVID-19 patients or if they are improving their outcomes.

**Figure 1:**
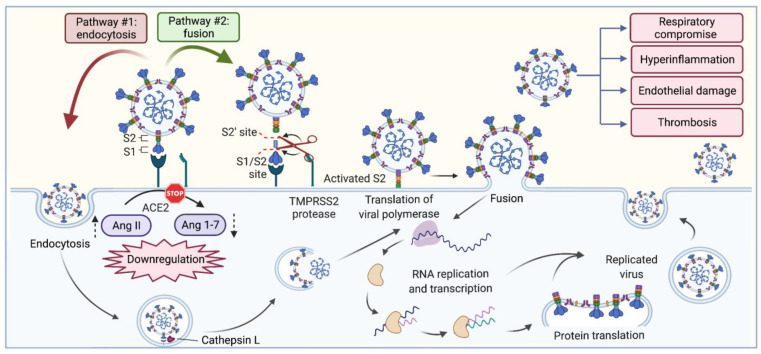
SARS-CoV-2 entry into the host cell. As an enveloped virus, SARS-CoV-2 can enter the host cell through one of two pathways—pathway #1, endosomal entry and pathway #2, direct cell surface entry. In both entry forms, the S protein binds to the ACE2 receptor and must be primed and activated by proteases at the S1/S2 and S2’ site. SARS-CoV-2 infection results in the downregulation of the ACE2 receptor, leading to an increase in Ang II and decrease in its conversion to Ang-(1-7). In pathway #1, SARS-CoV-2 undergoes clathrin-mediated endocytosis, and is primed by host protease Cathepsin L present in the endolysosome, followed by membrane fusion and release of viral genomic material. In pathway #2, the S protein is cleaved by TMPRSS2 at the cell membrane, where it directly fuses and releases viral RNA without entering an endolysosome. Both pathways join in the host cytoplasm, where a viral polymerase is translated, followed by RNA replication, structural protein translation, viral assembly, and release of progeny virus that continue to spread the infection to other cells. SARS-CoV-2 infection leads to respiratory compromise by causing lung injury, results in excessive inflammation with overactivation of leukocytes and production of inflammatory mediators, results in direct and inflammation-mediated damage to the endothelium, and induces a pro-thrombotic state. Reproduced with permission from Elsevier: Nitric Oxide, 128 (2022), 72–102 [71]. Abbreviations: ACE2, angiotensin-converting enzyme 2; Ang, angiotensin; S protein, spike protein; SARS-CoV-2, severe acute respiratory syndrome coronavirus 2; TMPRSS2, transmembrane serine protease 2.

SARS-CoV-2 is able to enter the cells through the membrane-bound ACE2 protein in which it uses the protein as a receptor [9] (Figure 1). ACE2 is a protein that exists in two forms: a transmembrane protein and a soluble form. The transmembrane protein form of ACE2 is found and expressed mainly in the type II pneumocytes within the alveolar epithelium of the lung, as well as in the epithelial cells of the intestine, kidney, and blood vessels [10]. Meanwhile, the soluble form of ACE2 is found in very low concentrations within the body but could be increased in pathological states such as hypertension and diabetes [11]. All of these locations could be potential entry points for the SARS-CoV-2 virus [53]. This binding between SARS-CoV-2 and ACE2 is accomplished through the virus’s spike protein. The spike protein associates with the extracellular domain of ACE2, stimulating a clathrin-dependent endocytosis of the entire complex and thus allowing entry into the cell [54]. Aside from the actual binding of the virus to the ACE2 receptor, SARS-CoV-2’s spike protein is also primed by the host serine protease transmembrane, serine protease-2 (TMPRSS2) before entry into the cell [55] (Figure 1). The membranes can fuse through this interaction between the spike protein and the TMPRSS2 protease, which further permits the virus to enter the body [56]. ACE2 cleaves angiotensin II into angiotensin (1-7), which has vasodilatory, anti-inflammatory, and antifibrotic effects through its binding with the Mas receptor [57]. Ang-(1-7) demonstrates opposing effects with that of angiotensin II when bound to the G-protein-coupled receptor Mas. Through this interaction, vasodilatory mediators, such as nitric oxide and prostaglandins, are released. In addition to vasodilatory effects, Ang-(1-7) also opposes angiotensin II’s vasoconstrictor and pro-inflammatory effects, which is seen when Ang II is bound to its receptor, AT1R [58]. Therefore, Ang-(1-7) could reduce inflammatory tissue damage and has been suggested as a potential treatment for the detrimental effects demonstrated by SARS-CoV-2 [59]. Essentially, ACE2’s function opposes the physiologic role of ACE and thus maintains a balance between pro-inflammatory and pro-fibrotic activity with anti-inflammatory and antifibrotic activity [60].

RAAS inhibitors affect the balance between pro and anti-inflammation and fibrosis, and there is an increased shift towards the pro-inflammatory and pro-fibrotic effects [61]. RAAS inhibition results in the upregulation of angiotensin-converting enzyme 2 (ACE2) expression on the cell surface, which allows the entry of SARS-CoV-2 [62]. As a result of this increased expression of ACE2, it may be likely that SARS-CoV-2 could enter the body more readily when there are more ACE2 receptors present [63]. Unfortunately, COVID-19 patients with underlying conditions such as hypertension and diabetes who are treated with RAAS inhibitors have been reported to have the highest rate of fatality by the COVID-19 virus [64]. These medications increase the virus’s severity and increase mortality by twofold [65]. Since hypertensive and diabetic patients already have a hyperactive Ang II-AT1 axis due to the pathologically overactive RAAS complex, when SARS-CoV-2 binds and downregulates the production of functional ACE2, it is more likely for these patients to develop severe conditions from the virus [66]. In these situations, if the body’s immune system is unable to fight against and defeat the infection, SARS-CoV-2 will be able to replicate after binding to ACE2 and further destroy the host’s cells. This results in an inability to inactivate Ang II’s signaling with its AT1 receptor and thus exacerbates lung infection and inflammatory mechanisms associated with angiotensin II [67]. Since SARS-CoV-2 binds to and downregulates ACE2, Ang II is not metabolized to Ang-(1-7); as a result, there are fewer anti-inflammatory and vasodilatory effects and more inflammation, fibrosis, oxidative stress, and vasoconstriction present [68].

Using RAAS inhibitors while treating COVID-19 is controversial because, aside from the harmful effects associated with their usage, data also show that ACE2 could have protective measures against COVID-19. After a person gets infected with COVID-19, their ACE2 levels diminish, which results in unregulated angiotensin II destroying tissues [10]. ACE inhibitors would be protective in this case because through RAAS inhibition, there would be a decreased concentration of angiotensin II, which promotes inflammatory responses and fibrosis within the lung among SARS-CoV-2 entry and binding to ACE2 [62]. This is so because, through the downregulation of ACE2 by SARS-CoV-2, the degradation of Ang II into Ang-(1-7) is decreased, which allows further destruction of tissues due to the increased levels of Ang II present in the body [55]. In addition, ARBs have been found to be beneficial in treating COVID-19 as well. This is due to ARB’s ability to upregulate ACE2 expression, resulting in increased levels of the protective mediator Ang-(1-7). Since ARBs directly inhibit angiotensin II’s activity on its receptor, they inhibit angiotensin II from its inflammatory and vasoconstricting effects [11]. Clinical trials have indicated another way in which RAAS inhibitors could provide protective effects against COVID-19; both ACE inhibitors and ARBs decrease the viral load and prevent peripheral T-cell depletion, which could help reduce the severity of the virus [69]. Although there may be either detrimental or protective effects associated with taking RAAS inhibitors while being treated for COVID-19, multiple societies such as the American College of Cardiology, the American Heart Association, and the International Society for Hypertension all agree that due to the inconclusive information and the current data available on the effects of RAAS inhibitors and COVID-19, patients taking these medications are not advised to change therapy and should continue with their normal treatment regimen [70].

## 4. Nitric Oxide and Its Function in COVID-19

Nitric oxide (NO) is one of three known gasotransmitters with critical roles in cardiovascular homeostasis and the immune response, earning it significant interest as a potential management option for COVID-19 [72,73,74,75]. It is endogenously produced from L-arginine by three isoforms of the enzyme nitric oxide synthase (NOS): neuronal NOS (nNOS or NOS-1), inducible NOS (iNOS or NOS-2), and endothelial NOS (eNOS or NOS-3) [76]. nNOS is known for its role in neurotransmission and is constitutively expressed in the body alongside eNOS, which is crucial in maintaining the vasculature through vasodilation and inhibition of platelet and leukocyte adhesion [77]. iNOS is an inducible isoform that becomes critical in host defense against pathogens [77]. eNOS and iNOS have received significant attention regarding their roles in COVID-19 severity [76,78,79,80]. Dysregulation of eNOS and decreased endothelial NO bioavailability can be seen in chronic inflammatory diseases such as hypertension and may be critical in predisposing individuals to higher severity of COVID-19 [81]. Dysregulation of iNOS can occur in a hyperinflammatory response to an invading pathogen such as SARS-CoV-2 and may lead to inflammatory damage mediated by excessive production of superoxide anions and peroxynitrite [82,83,84]. SARS-CoV-2 also contributes to NO deficiency via disruption of RAAS by downregulating ACE2 and angiotensin 1–7, a metabolite that promotes NO synthesis and release [58,85]. Additionally, COVID-19 has been linked to reduced arginine bioavailability, [86,87], and arginine supplementation has been found to reduce hospitalization duration with severe COVID-19 [88]. In individuals who already have compromised eNOS function, as would be the case for people with hypertension [83,84], infection with SARS-CoV-2, which causes further endothelial damage, hyperinflammation, and RAAS imbalance, may be particularly disastrous. Therefore, restoration of NO to such individuals may be highly beneficial for management and preventing progression of symptoms.

**Figure 2:**
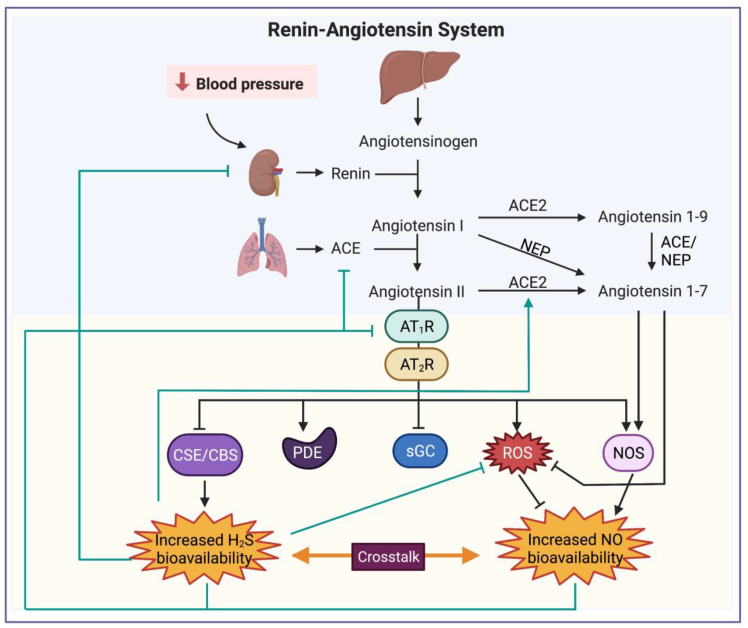
Interactions between RAAS, NO, and H_2_S. The RAAS consists of two main axes, the pro-inflammatory and vasoconstrictive ACE-Ang II pathway, and the anti-inflammatory, vasorelaxant ACE2-Ang-(1-7) pathway, both of which differently affect NO and H_2_S bioavailability. Ang II uses AT1R and AT2R to exert a variety of effects upon NO bioavailability; there are reports of Ang II increasing NOS activity while simultaneously increasing the production of ROS through NADPH oxidase, resulting in NOS uncoupling and a net decrease in NO bioavailability over time despite increased production and release. Ang II also inhibits sGC and increases the activity of some PDEs, diminishing the function of the NO signaling pathway. On the other hand, Ang-(1-7) augments NO bioavailability both by reducing ROS and by stimulating NOS activity. In return, NO downregulates ACE and AT1R, reducing RAAS activation. There are also reports of the RAAS affecting H_2_S bioavailability, where increasing Ang II decreased H_2_S bioavailability by downregulating CSE and CBS. Measures to increase H_2_S bioavailability reduce traditional RAAS activation, inhibiting or downregulating renin release, ACE, AT1R, and ROS levels while increasing ACE2 activity to increase the function of the anti-inflammatory axis of the RAAS. Both gasotransmitters also interact to reinforce the other’s increase in bioavailability. Thus, NO and H_2_S both act to reduce RAAS overactivation, serving as anti-inflammatory, antioxidant, and vasorelaxant mediators. Note: black arrows show effects of RAAS on NO and H_2_S, green arrows show effects of the gasotransmitters on components of RAAS. Abbreviations: ACE, angiotensin-converting enzyme; ACE2, angiotensin-converting enzyme 2; Ang, angiotensin; AT1R, angiotensin II type 1 receptor; AT2R, angiotensin II type 2 receptor; CBS, cystathionine β-synthase; CSE, Cystathionine γ-Lyase; H2S, hydrogen sulfide; NADPH oxidase, nicotinamide adenine dinucleotide phosphate oxidase; NEP, neutral endopeptidase; NO, nitric oxide; NOS, nitric oxide synthase; PDEs, phosphodiesterases; RAAS, renin–angiotensin–aldosterone system; ROS, reactive oxygen species; sGC, soluble guanylyl cyclase.

Before the establishment of infection, NO is of great interest in its capacity for early-stage prevention of COVID-19 infection. Studies using NO donors, experimental iNOS induction, and iNOS transfection have demonstrated a broad range of antiviral activity of both exogenously administered and endogenously produced NO [89,90,91,92,93,94,95,96,97,98,99,100,101]. These antiviral actions have been confirmed with SARS-CoV-2 (Figure 2), against which NO acts to inhibit viral replication and reduce viral load, demonstrated in vitro using NO donor S-nitroso-L-acetyl penicillamine (SNAP) and in a phase 2 clinical trial of nitric oxide nasal spray (NONS) [102,103]. Of note, synthetic dinitrosyl iron complexes, the innate version of which are important as storage and transportation for endogenous nitric oxide [104], have been identified as a promising protease inhibitor with potential in suppressing SARS-CoV-2 [105,106]. In individuals at higher risk of infection, including those with hypertension, the use of NO in the early stages may provide promise for preventing or curtailing SARS-CoV-2 infection.

**Figure 3:**
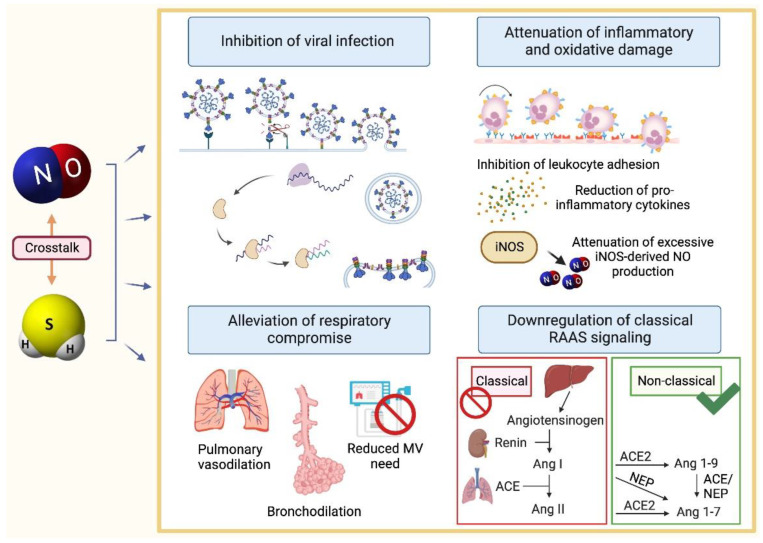
NO and H_2_S applications in COVID-19. NO and H_2_S are antiviral agents with promise for inhibiting SARS-CoV-2 infection through interference with entry, replication, and release. Both gasotransmitters also have potential applications in later-stage management of COVID-19. NO and H_2_S demonstrate the ability to inhibit leukocyte adhesion to the endothelium, reduce levels of pro-inflammatory cytokines, curtail excessive iNOS activity, and overall reduce inflammatory damage. These properties, combined with their vaso- and bronchodilator actions in the pulmonary system, serve to reduce the need for mechanical ventilation and alleviate respiratory compromise. Additionally, NO and H_2_S downregulate the classical RAAS signaling pathway and its related pro-inflammatory shift by inhibiting Ang II and AT_1_R. As a result, NO and H_2_S may have significant potential in the management of COVID-19, a disease recognized for causing hyperinflammatory conditions and respiratory compromise and for having greater severity where the RAAS is imbalanced. Abbreviations: Ang II, angiotensin II; AT_1_R, angiotensin II type 1 receptor; COVID-19; coronavirus disease 2019; H_2_S, hydrogen sulfide; NO, nitric oxide; RAAS, renin–angiotensin–aldosterone system; SARS-CoV-2, severe acute respiratory syndrome coronavirus 2.

NO is also a vasodilator, bronchodilator, and anti-inflammatory agent, which protects the vascular endothelium, thereby holding promise for managing later stages of COVID-19. As a broncho- and vasodilator, inhaled NO (iNO) is a potential rescue treatment for respiratory compromise (Figure 2) and has been found to improve oxygenation, reduce pulmonary vascular resistance, and decrease the need for invasive respiratory support among COVID-19 patients [107,108,109]. Additionally, NO therapies may prove important in containing the inflammatory damage responsible for the respiratory and cardiovascular injury that occurs in severe COVID-19 cases. Increasing NO bioavailability was found to curtail the production of pro-inflammatory cytokines, reduce leukocytic infiltration, and decrease neutrophil activation and adhesion in sepsis-induced neutrophils and in a rabbit model of lavage-induced ARDS [110,111] (Figure 2). Exogenously administered NO both restores NO to the endothelium, where it inhibits leukocyte adhesion and prevents further endothelial damage [112], and can exert negative feedback regulation on excessive iNOS activity [113], limiting the production of damaging byproducts such as superoxide anions and peroxynitrite [114] (Figure 2). Additionally, there is an extensive interplay between the RAAS and NO (Figure 3). Ang-(1-7), a component of the non-classical RAAS pathway, has been found to increase NO bioavailability by stimulating constitutive NOS activity [58,115,116] while curtailing excessive iNOS activity [117,118] and by reducing superoxide anion production (thereby attenuating NO consumption to form damaging products such as peroxynitrite) [119,120]. On the other hand, classical RAAS metabolite Ang II reduces NO signaling by inhibiting its receptor soluble guanylyl cyclase (sGC) [121], by increasing the breakdown of its downstream signaling molecule cyclic guanosine monophosphate (cGMP) through stimulation of phosphodiesterase 1A1 (PDE1A1) [122], and by promoting NOS uncoupling through increased production of superoxide anions from nicotinamide adenine dinucleotide phosphate hydrogen (NADPH) oxidase [123,124,125]. Classical RAAS overactivation thus would result in net decrease in the protective effects of NO, an effect that would be exacerbated by infection with SARS-CoV-2. Restoration of NO may thus prove a promising approach, and, in fact, there is evidence that NO inhibits ACE [126,127,128] and downregulates Ang II type I receptors (AT_1_R) [129,130], thereby reducing the activity of the pro-inflammatory arm of the RAAS. These actions are especially relevant in mitigating COVID-19 severity in individuals with an imbalance in the RAAS, who are indeed already predisposed to adverse outcomes from the virus. NO is also protective towards the vascular endothelium, which is especially important both in COVID-19 and its predisposing comorbidities such as hypertension which are notable for endothelial dysfunction [131]. Endothelial damage is characterized by insufficient eNOS activity and endothelial bioavailability of NO and is a major factor both in hypertension and in COVID-19 case severity [79]. Endothelial damage results in a shift to a prothrombotic and vasoconstrictive state that can be countered by restoration of NO to the endothelium. Augmentation of NO bioavailability using NO-releasing aspirin (NCX 4215) and enhancement of eNOS activity has in fact been shown to reduce neutrophil and platelet activation and aggregation in vitro and ex vivo [110,132]. In addition, nNOS-derived NO deficiency has been implicated in cardiomyocyte arrhythmogenesis [133], as has RAAS dysfunction [134]. It has been suggested that deficient NO-mediated S-nitrosylation of ryanodine receptors potentially mediates calcium-triggered ventricular arrhythmias [133,135]. Thus, it is possible that this additional comorbidity may benefit from NO therapies in the context of RAAS dysfunction and COVID-19.

The ability of NO to prevent and limit infection, ameliorate pulmonary complications, resolve inflammation, restore RAAS balance, and protect the endothelium makes NO therapy an approach worth investigating for prevention and later-stage management of COVID-19. Hypertensive and diabetic patients, as discussed previously, are at increased risk of severe COVID-19 infection, are more susceptible to the accompanying hyperinflammatory response, and come with a history of existing endothelial damage. In these patients, using NO therapies, including iNO, NO donor molecules, and even dietary nitrates for prevention, may be a promising option.

## 5. Hydrogen Sulfide and Its Function in COVID-19

H_2_S was the last of the gasotransmitters to be discovered. It shares many of its properties with those previously discussed for NO—an antiviral, anti-inflammatory agent protective towards the lungs and vascular endothelium [136]. H_2_S is endogenously produced from the amino acid L-cysteine by a group of four enzymes constituting three enzymatic pathways: cystathionine γ-lyase (CSE), cystathionine β-synthase (CBS), and 3-mercaptopyruvate sulfurtransferase (3-MST) in conjunction with cysteine aminotransferase (CAT) [137]. Although underexplored in comparison to NO, the evidence suggests that H_2_S therapy may be an avenue worth investigating for future use.

H_2_S has a broad range of antiviral activity against enveloped RNA viruses belonging to orthomyxoviridae, filoviridae, flaviviridae, bunyaviridae, and paramyxoviridae families through inhibition of viral entry, replication, and assembly/release [138,139], properties that may transfer to SARS-CoV-2 (Figure 2). H_2_S administration using both fast and slow H_2_S donors has also been reported to downregulate the expression of TMPRSS2 in human airway epithelial cells [140], which is vital in the entry of SARS-CoV-2 into the host cell [56]. By this mechanism, H_2_S may prevent SARS-CoV-2 infection of the host. In addition, upregulation of glutathione (GSH) by H_2_S [141] may be another mechanism of inhibiting SARS-CoV-2 infection through both its antioxidant properties and possibly direct interference with the actions of the ACE2 and TMPRSS2 proteins [142]. N-acetylcysteine (NAC) is a compound that yields GSH [143] as well as increased H_2_S levels [144], and has been suggested as a therapy for the management of COVID-19 [145]. In one study, while NAC was unsuccessful in inhibiting cell fusion of SARS-CoV-2, its stronger derivative NACA inhibited S protein binding to ACE2 and viral entry in vitro and in mice transfected with human ACE2 and TMPRSS2 [146].

Later-stage management of COVID-19 symptoms may also be possible using H_2_S therapies, owing to its actions as a bronchodilating, anti-inflammatory, and endothelium protective agent. H_2_S donors have demonstrated the ability to increase oxygenation, ameliorate ALI, and reduce VILI in models of pulmonary damage [147,148] (Figure 2). These findings appear to translate to studies with COVID-19 patients administered with NAC, which have demonstrated improved oxygenation, reduced lung damage and inflammatory markers, and decreased need for respirators [149,150]. Additionally, as already mentioned, SARS-CoV-2 infection downregulates the ACE2 protein [151], which disturbs the balance between the pro- and anti-inflammatory arms of the RAAS, causing a shift to a vasoconstrictive state and resulting in pulmonary damage [152,153]. Restoration of ACE2 after infection may be significant in reducing inflammatory damage and protecting against lung injury, having demonstrated these protective actions in one murine model of sepsis-induced severe acute lung injury [154]. In fact, Ang II has been found to inhibit CSE [155]; therefore, restoring ACE2 and reducing Ang II levels may also serve to restore the protective function of H_2_S (Figure 3). Considering the findings of one study in which administration of NaHS upregulated ACE2 expression in the endothelial cells of atherosclerotic mice [156], it is possible that H_2_S administration post-infection with SARS-CoV-2 may restore the RAAS balance and enforce its own restoration and correspondingly play a role in protecting against lung injury. Additionally, there is evidence that H_2_S attenuates the pathological overactivity of the classical RAAS pathway [157] by inhibiting ACE activity [158], downregulating AT_1_R [159,160], and inhibiting renin activity [161] (Figure 3). Thus, restoration of H_2_S may work against the inflammatory and vasoconstrictive shift that occurs both in hypertension and in COVID-19. H_2_S also has a protective function in the vascular endothelium, making it important for vascular complications arising from endothelial dysfunction. H_2_S contributes to vasodilation, inhibits leukocyte and platelet adhesion to the endothelium, and increases NO bioavailability in the endothelium by increasing eNOS activity [162] (Figure 2). H_2_S depletion yields endothelial dysfunction, and restoration of H_2_S to the deficient endothelium can decrease thrombosis while increasing thrombolysis and reducing endothelial cell apoptosis [163,164,165,166]. H_2_S also demonstrates crosstalk with NO, curtailing excessive iNOS activity [167,168] while upregulating eNOS activity [169,170] and reducing superoxide levels, further increasing NO bioavailability [171] (Figure 3). This action provides further reason to investigate H_2_S use in COVID-19 patients, considering the dysregulation of both NOS isoforms that may play a role in severe COVID-19 cases.

Similar to NO, H_2_S shows promise in the prevention and in management of severe COVID-19 infection. Taking advantage of extensive feedback between the two gasotransmitters may also be a possibility when considering potential therapies. Making these therapies available to the general public may be especially important in reducing the rate of negative outcomes among populations already vulnerable to the burden of the pandemic.

## 6. Racial Disparities and COVID-19 Outcomes

Hypertension has been highly prevalent among the black community [172]. It is hypothesized that the black population faces a higher incidence of hypertension than other races because of increased stress and lack of compliance to medication seen within this population [173]. Although hypertension is more prominent among black people, there is data demonstrating that some antihypertensive drugs, such as ACE inhibitors and ARBs, are less effective and have increased consequences when given to black people than to individuals of other races [174]. Some reasons this is the case in blacks and not in other races could be genetic variants and mutations. For example, many ARBs are metabolized through the cytochrome P450 2C9, and for some reason, that cytochrome is less common in blacks than in whites [175]. In addition, there is data showing that blacks have naturally low renin levels due to genetic mutations, making ACE inhibitors and ARBs more resistant in this population due to RAAS deactivation through its negative feedback mechanism [176]. Due to these reasons, not only are ACE inhibitors and ARBs less effective in blacks, but they have been shown to have adverse effects as well. A recent cohort study evaluating the cardiovascular outcomes associated with ACE inhibitor use among white and black patients has demonstrated that the black patients taking the ACE inhibitors dealt with increased cardiovascular outcomes, such as nonfatal myocardial infarction and nonfatal stroke, compared to white patients [177]. Another cohort study observed the outcomes in black hypertensive patients taking ACE inhibitors and compared them to other antihypertensive drugs, such as calcium channel blockers (CCB), thiazide diuretics, and beta blockers. This study showed an increase in myocardial infarction, stroke, and death among the black hypertensive patients taking ACE inhibitors compared with the patients who took CCB, thiazide diuretics, or beta-blockers [178]. Not only are cardiovascular outcomes an adverse effect in black patients taking ACE inhibitors and ARBs, but black patients are also more likely to experience angioedema when taking ACE inhibitors than other races. Since the mechanism behind angioedema is primarily a result of increased bradykinin levels, ACE inhibitors are the drugs associated with this side effect, not ARBs [179]. A prospective study demonstrated that ACE inhibitors increase the risk of developing angioedema in blacks by threefold compared with whites [180].

Ethnic minorities have been one of the groups to have been hit the hardest by the COVID-19 virus. Several factors, such as lifestyle conditions, have contributed to this cause. Chronic diseases such as diabetes, hypertension, and obesity are more prevalent in black people than in white people, and unfortunately, these conditions have been associated with increased severity of COVID-19. Not only are chronic health conditions more common in ethnic minorities, but these populations are more likely to live in crowded areas, use more public transportation, and work jobs that pay less and do not provide sick days [181]. The ethnic minority group, especially in the United States, makes up a significant portion of “essential workers”, which includes supermarket workers, health-care workers, and public transportation workers [182]. All of these factors increase the likelihood of being exposed to the virus. Aside from these lifestyle factors that have been linked with an increase in COVID-19 exposure amongst this demographic of people, medication, such as RAAS inhibitors, could also play a role in acquiring this virus. Even though ACE inhibitors and ARBs are contraindicated as first-line monotherapy treatments in blacks, they are still used in a combination regimen with other antihypertensive drugs such as calcium channel blockers and thiazide diuretics [174]. Since RAAS inhibitors are being used as a combination therapy, they still contribute to the negative side effects seen in black people. Not only would they contribute to adverse effects, but RAAS inhibitors even in combination with other drugs, would continue to up regulate the ACE2 protein, which would increase the risk of the SARS-CoV-2 virus entering the body According to statistics from April 2020, the ethnic minority population makes up the most cases of COVID-19 in both Europe and the United States. Based on the data from the UK’s Intensive Care National Audit and Research Centre, about 5000 people were severely ill in April from COVID-19 in England, Wales, and Northern Ireland. Of those 5000 cases, 34% of the cases were from a minority background even though those ethnic groups only make up about 14% of the total population within those regions. Meanwhile, in the United States, according to the data released by the CDC from 18 April 2020, among 120,000 cases of COVID-19, 36% of those cases were non-white people, who make up around 23% of the United States population. Most cases were by the black and African American groups, who made up 30% of the cases even though they only represented 13% of the total population [183].

## 7. Summary and Perspectives

H_2_S is a gas that has various physiological functions and benefits, including cell survival and anti-inflammatory actions [184]. Studies show that H_2_S could portray protective properties against COVID-19. It has been demonstrated that H_2_S can suppress the transcription of TMPRSS2, the protease involved in the priming of the coronavirus’ spike protein to allow cell entry [185]. H_2_S also inhibits RNA viral replications within the lungs of several RNA viruses, such as the influenza virus and Ebola [138]. Therefore, it suggested that H_2_S could inhibit the coronavirus’ RNA viral replication. H_2_S’s mode of action for inhibiting these viral replications is through the downregulation of the viral proteins and the syncytium formation and virus assembly [139]. Exogenous H_2_S has protective properties in the lungs and has been effective in reversing lung damage and inflammation, as seen in COVID-19. Exogenous H_2_S could be given through N-acetylcysteine (NAC), a supplemental form of the amino acid cysteine. NAC is not only an antioxidant but also releases H_2_S and can be used to protect organs, such as the lungs, heart, and kidneys, from COVID-19’s cytokine storm [186].

NO, a vasodilator produced by endothelial cells, is another mediator that could potentially be used to treat COVID-19. NO synthesis and release are promoted by Ang-(1-7), a metabolite produced by ACE2 [187]. Since SARS-CoV-2’s spike protein binds to ACE2, it affects the production of nitric oxide through the decreased production of Ang-(1-7) [72]. Therefore, exogenous nitric oxide could benefit this situation because of its anti-inflammatory effects. NO has been associated with protective measures, especially in patients with acute respiratory distress system (ARDS), which is often a consequence of the COVID-19 virus. Not only is nitric oxide able to inhibit the cytokine storm present in the coronavirus, but it also improves oxygenation of the arteries and inhibits pulmonary hypertension through its vasodilatory actions within the lungs [188]. Supplemental nitric oxide could be administered through several routes, such as inhaled nitric oxide and donor compounds, to help combat the detrimental effects of this virus [189]. Studies have shown that the nitric oxide donor, S-nitroso-N-acetylpenicillamine (SNAP), has been able to interfere with the fusion of the SARS-CoV spike protein with its receptor as well as diminish the virus’ RNA synthesis [75].

It is crucial to have other options available for treating and potentially preventing COVID-19. Although vaccines are available since they are so new, their efficacy and long-term side effects are still unknown. Therefore, aside from unknown side effects from the COVID-19 vaccines, it would be essential to have alternative options for preventing and treating this virus, especially in individuals who refuse to receive the vaccine, to ensure their protection.

## Abbreviations

3-MST	3-mercaptopyruvate sulfurtransferase
ACE2	angiotensin-converting enzyme 2
ALI	acute lung injury
Ang	angiotensin
ARB	angiotensin receptor blockers
ARDS	acute respiratory distress syndrome
CAT	cysteine aminotransferase
CBS	cystathionine β-synthase
cGMP	cyclic guanosine monophosphate
COPD	chronic obstructive pulmonary disease
COVID-19	coronavirus disease 2019
CSE	cystathionine γ-lyase
eNOS	endothelial nitric oxide synthase
GSH	glutathione
H_2_S	hydrogen sulfide
iH_2_S	inhaled hydrogen sulfide
IL	interleukin
iNO	inhaled nitric oxide
iNOS	inducible nitric oxide synthase
MV	mechanical ventilation
NAC	N-acetylcysteine
nNOS	neuronal nitric oxide synthase
NO	nitric oxide
PDE5	phosphodiesterase 5
RAAS	renin–angiotensin–aldosterone system
ROS	reactive oxygen species
S protein	spike protein
SARS	severe acute respiratory syndrome
SARS-CoV-2	severe acute respiratory syndrome coronavirus 2
sGC	soluble guanylyl cyclase
SNP	sodium nitroprusside
SOD	superoxide dismutase
TMPRSS2	transmembrane serine protease 2
